# Transconjunctival suprachoroidal buckling for rhegmatogenous retinal detachment

**DOI:** 10.1186/s40942-025-00774-2

**Published:** 2025-12-12

**Authors:** Ehab N. El Rayes, Ahmed Al Tayyar, Stratos Gotzaridis, Yi-Ting Hsieh

**Affiliations:** 1 Retina Department, Research Institute of Ophthalmology (RIO), Giza, Egypt; 2 My Retina Athens Eye Center, Athens, Greece; 3 Department of Ophthalmology, National Taiwan University, Taipei, Taiwan; 4 Department of Ophthalmology, National Taiwan University Hospital, Hsin- Ch Branch, Hsinchu, Taiwan

**Keywords:** Suprachoroidal space, Suprachoroidal buckle, In-office suprachoroidal buckle for RRD

## Abstract

**Background:**

To assess the effectiveness of the transconjunctival suprachoroidal buckle (TSCB) technique in treating primary rhegmatogenous retinal detachment (RRD).

**Methods:**

A prospective interventional study including patients with primary RRD undergoing the TSCB technique. The technique consisted of a transconjunctival approach using an olive tip handled cannula with an Atkinson 25-gauge needle tip that injects a high-purity gel in the suprachoroidal space (SCS) and creates a buckle effect that lasts for 12–18 months. Indirect laser retinopexy is done in the operating room or in the early post-operative office visit on the slit lamp. The TSCB technique could be done in the office in selected cases of uncomplicated RRD.

**Results:**

The study included 31 eyes of 31 patients. Seventeen eyes were phakic (55%). The RRD involved one quadrant in 81% of eyes. Sixteen eyes (52%) had more than one break. The TSCB procedure was performed in the operating room in 21 patients (68%). The mean duration of follow-up was 5 months. Postoperatively, we achieved retinal attachment in all patients. Three patients (10%) needed a second surgery. Two patients (6%) developed dot hemorrhage due to choroidal puncture.

**Conclusion:**

The TSCB is safe, and avoids the complications of conventional scleral buckling.

**Supplementary Information:**

The online version contains supplementary material available at 10.1186/s40942-025-00774-2.

## Background

The key to the success of retinal detachment surgery is creating a chorioretinal adhesion that approximates the choroid and retinal pigment epithelium (RPE) to the detached retina. The choroid at the site of the retinal break is treated with cryotherapy intra-operatively or laser post-operatively to create the desired chorioretinal adhesion. There are 3 surgical approaches to achieve retinal re-attachment in patients with uncomplicated retinal detachment. These are pneumatic retinopexy, scleral buckle surgery, and pars plana vitrectomy (PPV). Each of these three techniques has a high success rate but with inherent potential complications related to their invasiveness [1–4]. Several authors attempted to induce a localized iatrogenic choroidal detachment using air or gas to produce an indentation that seals the retinal break through a suprachoroidal approach [5–7]. In 2013, we described the technique of suprachoroidal buckling using a catheter to inject 20 mg/ml hyaluronic acid (HA) (Perlane, Galderma; off-label use) to close retinal tears and treat retinal detachment [8]. This was followed by several publications on this technique using a 23-gauge suprachoroidal cannula (MedOne Surgical), and looking into different fillers including Healon 5 (Johnson and Johnson) to use in this technique. All necessitate doing a small sclerotomy to deliver the filler [9–11]. We describe a modification of our original technique that consists of a transconjunctival suprachoroidal buckle (TSCB) approach without the need for a sclerotomy. The rationale of the study is to assess the effectiveness of the TSCB technique in treating primary rhegmatogenous retinal detachment (RRD).

## Patients and methods

This is a prospective interventional study including consecutive patients with primary RRD. Inclusion criteria included primary RRD including patients presenting with proliferative vitreoretinopathy (PVR) grade A and B. The study excluded patients with PVR grade C or more, RRD with high-risk of developing PVR such as aphakia, trauma, and associated choroidal detachment. In addition, the study excluded patients with recurrent RRD or history of previous PPV, patients with associated macular hole, and patients with MTM. Preoperative examination included visual acuity assessment using the decimal notation, slit-lamp examination to assess the status of the crystalline lens, and ultrasonography to assess the status of posterior vitreous detachment (PVD). We performed retinal examination using the slit-lamp biomicroscopy (+ 90D lens) and indirect ophthalmoscopy including scleral indentation to determine the location and number of retinal breaks, retinal quadrants involved, and grade of PVR. Preoperative examination included fundus photo for documentation and optical coherence tomography (OCT) scan to exclude the presence of a macular hole.

**Fig. 1:**
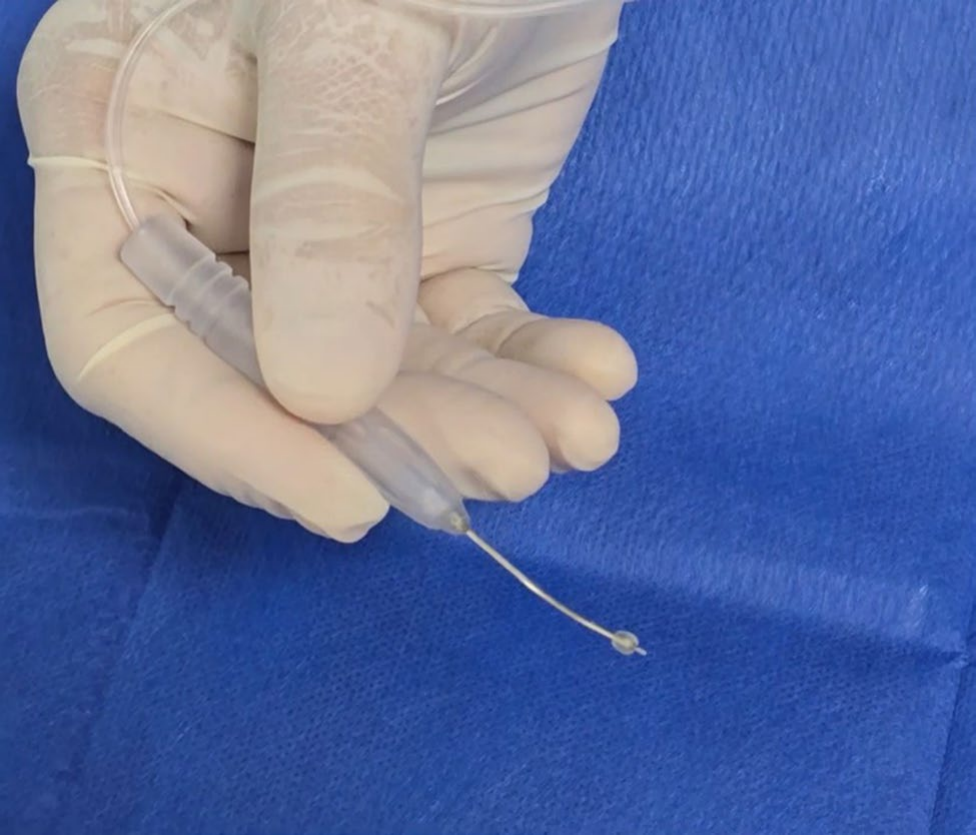
The Atkinson 25-gauge needle with an olive tip cannula used in the TSCB technique

**Fig. 2:**
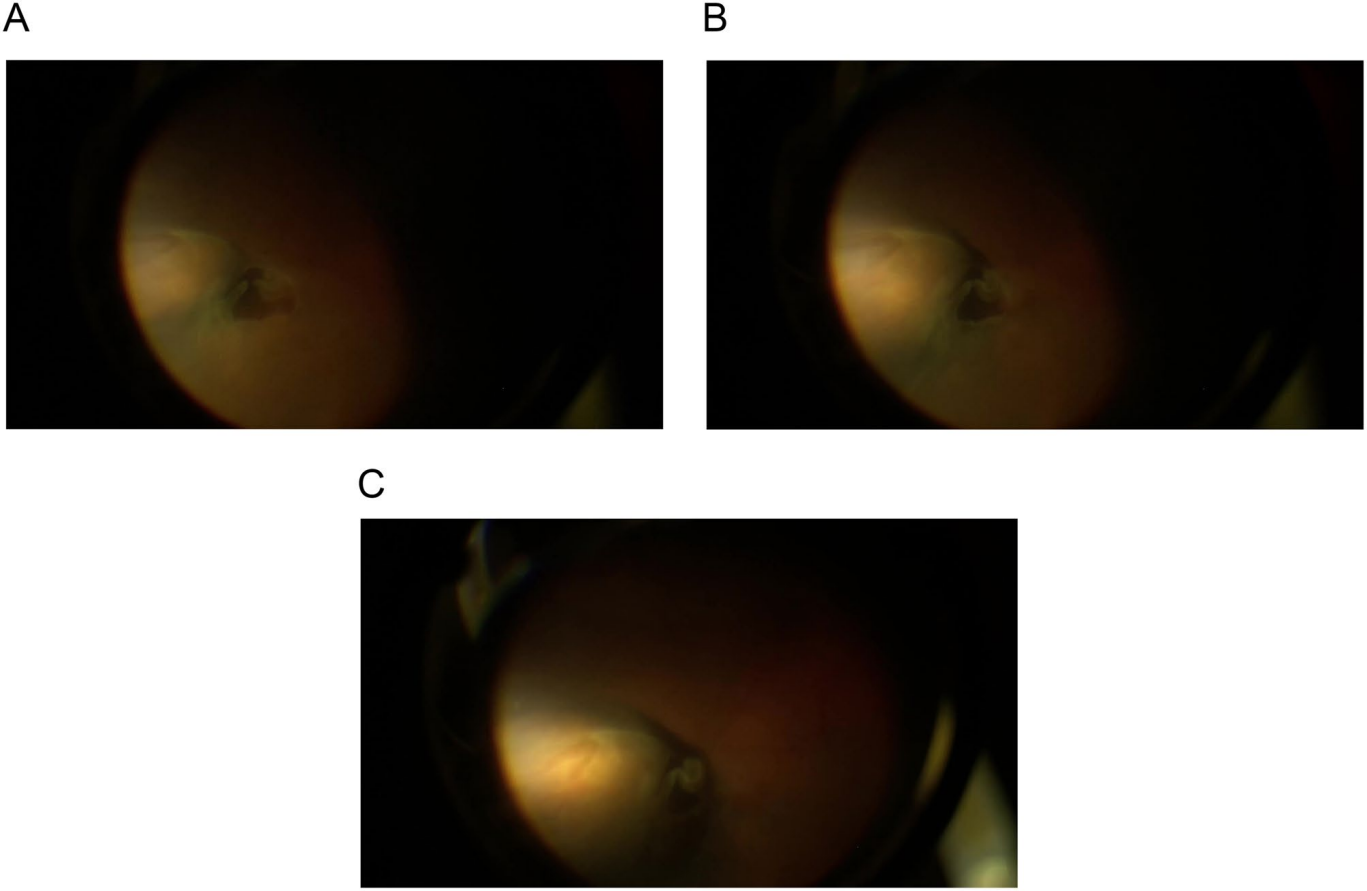
Intra-operative photos showing stepwise progression of the expanding buckle effect from 2 A through 2 C as the Perlane is injected in the suprachoroidal space

The surgical technique consisted of a transconjunctival approach using an olive tip handled cannula with an Atkinson 25-gauge needle tip. Figure 1. The needle is 2 mm in length. The olive tip is used as scleral depressor to localize the break prior to injection and as a guard by resting on the conjunctiva and sclera. This technique is done under local anesthesia using the indirect ophthalmoscope or chandelier-assisted using the surgical microscope. The cannula has a high- pressure tubing connected to a syringe containing 20 mg/ml hyaluronic acid (HA) (Perlane; Q-med, Uppsala, Sweden). Perlane is a high-purity gel containing stabilized non-animal HA that creates a long-lasting three-dimensional gel network lasting 12–18 months in the SCS as shown in our previous work. It is an inert substance that we used safely in previous studies without any tissue reaction. Other substance as Healon 5 or Healon GV could also be used but lasts 10–14 days based on our previous trials [10–12]. First, the surgeon gently slides the olive tip cannula over the conjunctiva to examine and localize the retinal breaks. Once the break is localized the surgeon rotates the cannula so the tip with the Atkinson needle is pointing toward the sclera and gently pushes the needle while observing the dimpling of the choroid as the needle punctures the sclera. Once the dimpling is in the correct site at the center of the retinal break, the surgeon injects the filler to gradually elevate the choroid and create the buckling effect in the desired height and width. Figures 2A-C. The injected volume differs according the break size and the amount of subretinal fluid. Usually, 0.1–0.3 ml is sufficient to close a single break. This technique could be used to closed multiple breaks in different quadrants by repeating the injection in selected sites. An Anterior chamber tap is needed to adjust the intraocular pressure (IOP). **SDC 1**. Indirect laser retinopexy could be done in the operating room if the break settles on the choroid or could be done in the early post-operative office visit on the slit lamp. The TSCB technique could be done in the office in selected cases of uncomplicated RRD. All surgical procedures were done in a retina tertiary center by a single vitreoretinal surgeon (EE). The main outcome measures were retinal re-attachment rate and complications related to the TSCB technique.

## Results

The study included 31 eyes of 31 patients. Sixteen patients were females (52%). Mean age was 55 years, (range 15–76 years; SD 13). Mean preoperative best-corrected visual acuity (BCVA) was 0.02 decimal units (range 0.001-0.2; SD 0.04). Seventeen eyes were phakic (55%). The retinal detachment involved one quadrant in 25 eyes (81%) and two quadrants in six eyes (19%). Fifteen eyes (48%) had two breaks, 15 eyes (48%) had a single break, and one eye (3%) had 3 breaks. The retinal break was located in the inferior quadrants in 13 eyes (42%), and in the superior quadrants in 13 eyes (42%). Five eyes (16%) had retinal breaks in the superior and inferior quadrants. The TSCB procedure was performed in the operating room in 21 patients (68%). The mean duration of follow-up was 5 months (range 0.5–13 months; SD 4). Postoperatively, we achieved retinal attachment in all patients (100%), of whom 28 patients (90%) had retinal re-attachment after a single procedure, and 3 patients (10%) needed a second surgery. Those latter patients were operated during the initial phase of the study. The mean postoperative BCVA was 0.4 decimal units (range 0.1–0.7; SD 0.2). Two patients (6%) showed dot hemorrhage at the site of choroidal dimpling due to choroidal puncture by the 25 g needle. In these two cases the needle was retracted and the injection done to complete the procedure successfully.

## Case presentation

**Fig. 3:**
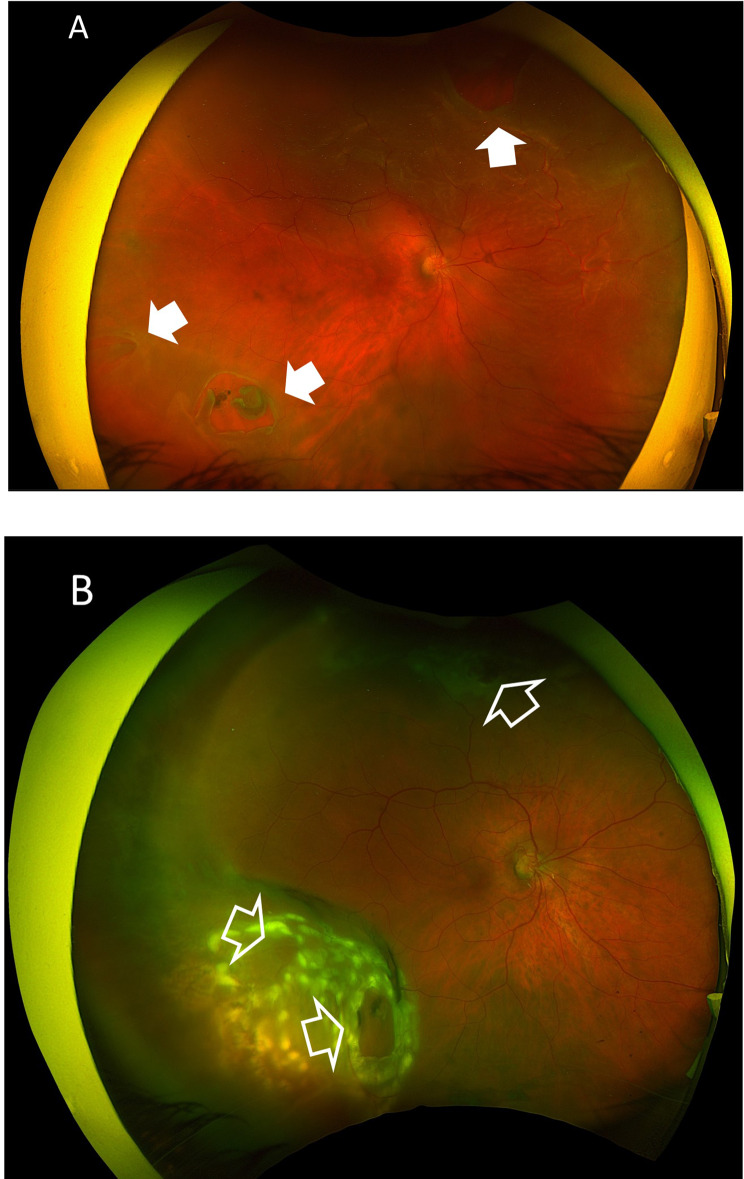
(**A**) A pre-operative color fundus photo of the right eye of a 55-year-old female patient with subtotal RRD. She had 2 retinal breaks anterior to the equator in the infero-temporal quadrant, and a third break in the supero-nasal quadrants (white arrows). (**B**) Color fundus photo of the same eye after the TSCB technique showing an attached retina and adequate laser retinopexy of the retinal breaks (open arrows)

Case # 1. A 55-year-old female patient presented with a sudden onset of veiling of the visual field in the right eye for 2 days. Her BCVA was hand motion (HM). The crystalline lens was clear and in place. Fundus examination revealed a subtotal RRD. She had 2 retina breaks in the infero-temporal quadrant and a single break in the supero-nasal quadrant. We performed the TSCB technique in the outpatient that resulted in a successful reattachment of the retina. We did laser retinopexy using a laser indirect ophthalmoscope (LIO). Figures 3A-B.

**Fig. 4:**
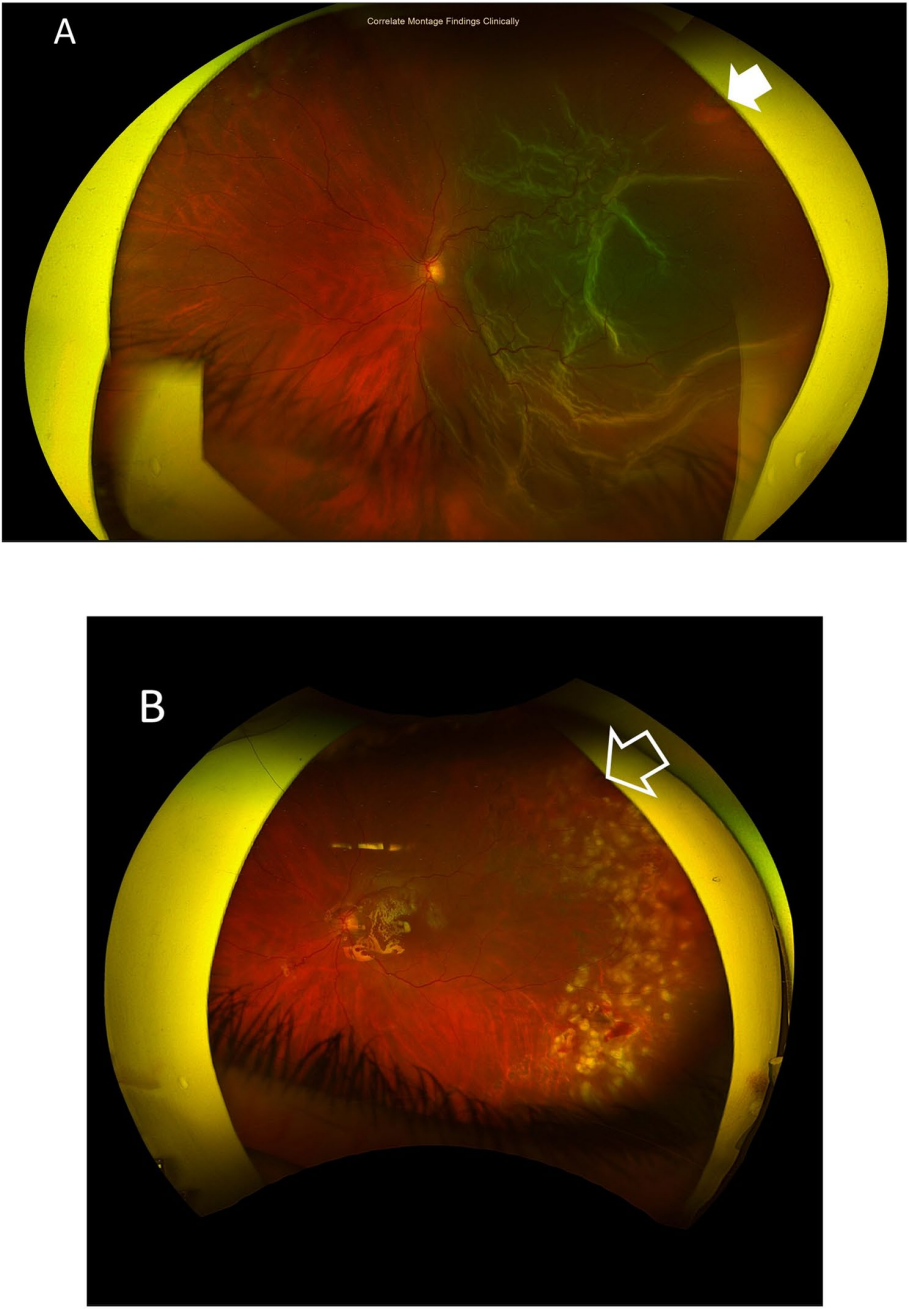
**A**) A pre-operative color fundus photo of the left eye of a 39-year-old female patient with subtotal RRD. She had a single pre-equatorial retinal break in the supero-temporal quadrant (white arrows). **B**) Color fundus photo of the same eye after the TSCB technique showing an attached retina and adequate laser retinopexy of the retinal break (open arrows)

Case # 2. A 39-year-old female patient presented with a sudden diminution of vision in the left eye for 4 days. Her BCVA was 0.1. The crystalline lens was clear and in place. Fundus examination revealed a subtotal RRD. She had a single retinal break in the supero-temporal quadrant. We performed the TSCB technique in the outpatient that resulted in a successful reattachment of the retina. We did laser retinopexy the following day using slit-lamp laser delivery. Figures 4A-B.

## Discussion

We have shown the concept of suprachoroidal buckling using a sclerotomy with successful results in several publications [8–12]. Boden et al. [13] showed similar success using a suprachoroidal hydrogel buckle. The conventional technique included a conjunctival incision and a small sclerotomy to inject the hydrogel, and the use of diathermy to cut-down the last scleral lamellae to expose the choroid. Therefore, it necessitated the use of the operating room and was performed as a surgical procedure. In this novel technique we are using a transconjunctival approach to create the suprachoroidal dome and close the retinal breaks in a minimally-invasive surgical procedure or even a potential in-office procedure. Depending on the nature and complexity of the detachment we have to decide if this patient is legible for an in-office TSCB or needs the surgical setup in the operating room, for instance in patients with multiple breaks and a large amount of subretinal fluid that requires drainage. Chorioretinal adhesion could be created by cryotherapy prior to injection of the filler, or using an indirect ophthalmoscope laser after creating the suprachoroidal buckle, or using the slit-lamp laser delivery the day after creating the suprachoroidal buckle and after resolution of the sub-retinal fluid. Our procedure is minimally-invasive, nonetheless it requires a learning curve to estimate the force needed to puncture the sclera with the Atkinson needle tip and dimple the choroid atraumatically prior to starting the injection. The blunt olive bulb surrounding the needle is used as a scleral depressor for the localization of the retinal break and at the same time a guard that rests on the scleral surface while performing the injection. The variation in the thickness of the sclera from one patient to another and across different quadrants poses a significant challenge, yet the direct visualization of the tip of the needle underneath the choroid helps the surgeon to judge the correct localization of the needle under the choroid and in relation to the retinal break. The 25-g Atkinson needle provides resistance to puncturing the sclera, this acts as a protective mechanism for the choroid. An additional challenge is location of the retinal tear underneath a rectus muscle, which necessitates puncturing the muscle and the sclera. In this situation, we use a longer 2 mm needle. Direct visualization of the tip of the needle while doing the indentation is the key for a successful procedure and is seen as a dimpling effect in the choroid once the puncture of sclera is achieved. The nature of the filler used depends on the complexity of the case. For a simple fresh retinal break we can either use Healon 5TM 2.3% (Johnson and Johnson), or Healon GV Pro 1.8% (Johnson and Johnson). The latter formula lasts 10 to 14 days in the suprachoroidal space until chorioretinal adhesion is achieved. We prefer using a highly cross-linked 20 mg/ml sodium hyaluronate; Perlane (Galderma) for larger breaks (off label use) and in situations when we require a longer buckle duration from 12 to 14 months in the SCS. In our opinion, this is the best option to get a good buckle height for sufficient amount of time even after chorioretinal adhesion occurred. Therefore, we recommend using Perlane in the initial learning period. Since this procedure is done transconjunctivally, we can buckle more than one break in different quadrants in the same procedure through multiple injection sites in cases with RRD with multiple breaks. Knowledge of the complexity of the retinal detachment, the amount of subretinal fluid, and the tolerance of the patient is important to decide which patient could be done in-office or in a minor operating room.

## Conclusion

The TSCB technique is a new minimally-invasive option in treating retinal detachment. The technique is safe, reproducible, and avoids some of the potential complications of scleral buckling in management of retinal detachment. We recommend that beginning surgeons select patients with clear media, well-dilatable pupil, and a single well-localized retinal break.

## Supplementary Information

Below is the link to the electronic supplementary material.

Supplementary Material 1: SDC 1. A video showing the TSCB technique for buckling superior and inferior breaks in a patient with rhegmatogenous retinal detachment

## Data Availability

All data about the present study are confidential. Access to these data will be granted exclusively to people or entities who meet the criteria for access to confidential data and only upon written request. All requests should be addressed to the corresponding author: Professor Ehab N. El Rayes. 35 Salah Salem St., (El Borg), Suite 702, El-Obour bldg. Cairo 11371, Egypt.
